# Correction: Microfluidic-based imaging of complete *Caenorhabditis elegans* larval development

**DOI:** 10.1242/dev.200126

**Published:** 2021-09-13

**Authors:** Simon Berger, Silvan Spiri, Andrew deMello, Alex Hajnal

There was an error in Development (2021) **148**, dev199674 (doi:10.1242/dev.199674).

The Movies appeared in the wrong order in the in-text links and the Supplementary Information, and so did not correspond with the legends, which were in the correct order:

Movie S2 was shown as Movie S1

Movie S3 was shown as Movie S2

Movie S4 was shown as Movie S3

Movie S5 was shown as Movie S4

Movie S6 was shown as Movie S5

Movie S1 was shown as both Movie S6 and S7

The Supplementary Information and in-text links have been corrected and the Movies are now shown in the correct order alongside the correct legend.

We apologise to the authors and readers for this error and any inconvenience it may have caused.

