# Supplementary material for: Microfluidic-based imaging of complete Caenorhabditis elegans larval development
Source: Development. 2021 Jul 21;148(18):dev199674. doi: 10.1242/dev.199674 (PMC8327290; doi:10.1242/dev.199674)
Supplement: Supplementary information [file develop-148-199674-s1.pdf]

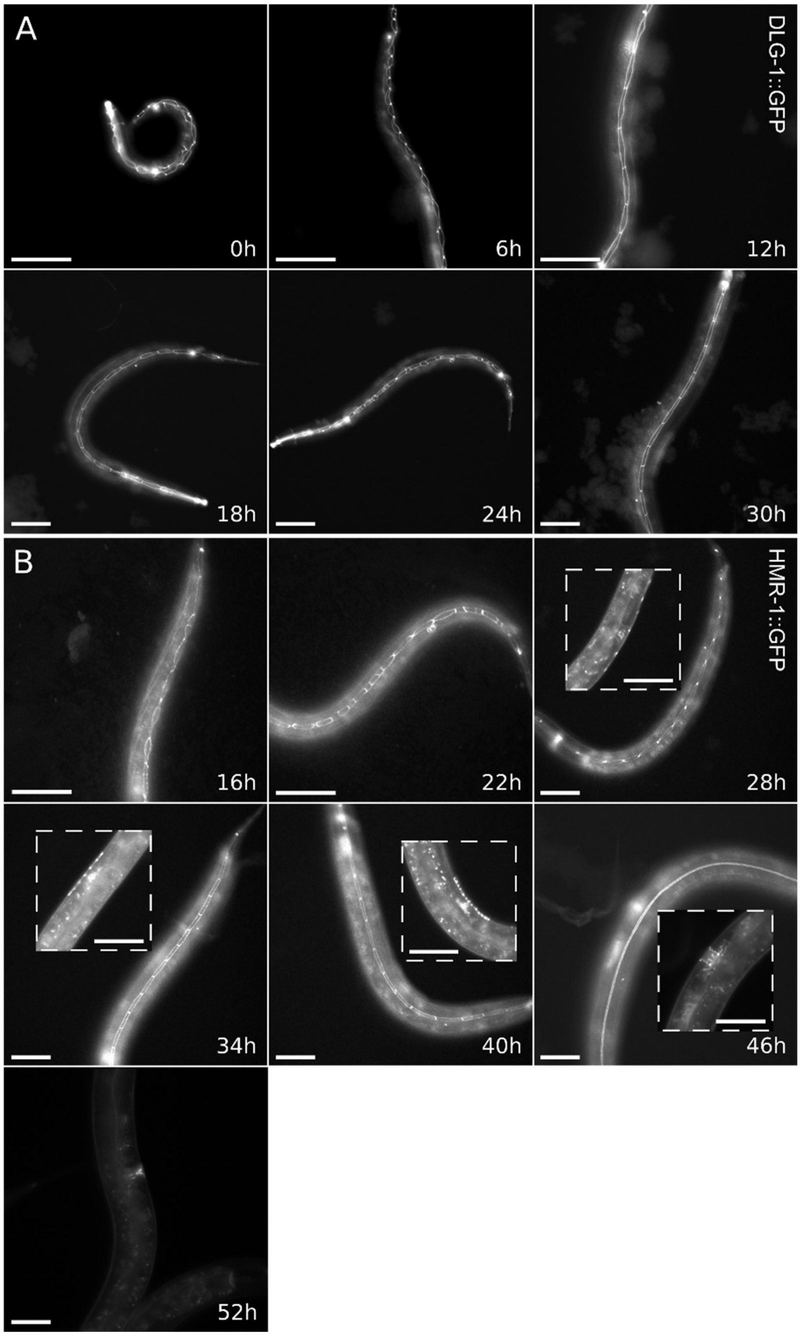

Fig. S1. Control of developmental timing on NGM plates. Representative images of worms with either the (**A**) DLG-1::GFP or (**B**) HMR-1::GFP. (**A**) Seam cells visualized in DLG-1::GFP expressing animals were imaged from overnight starved L1s (t = 0h) up to the end of L2 stage (t = 30h). (**B**) Seam cells in HMR-1::GFP expressing animals from 16 hours post seeding of overnight starved L1 animals up to the L4/adult transition indicated by vulval eversion. Dashed insert shows a view of the VPCs/vulva at the specific developmental stage. All scale bars 50 µm.

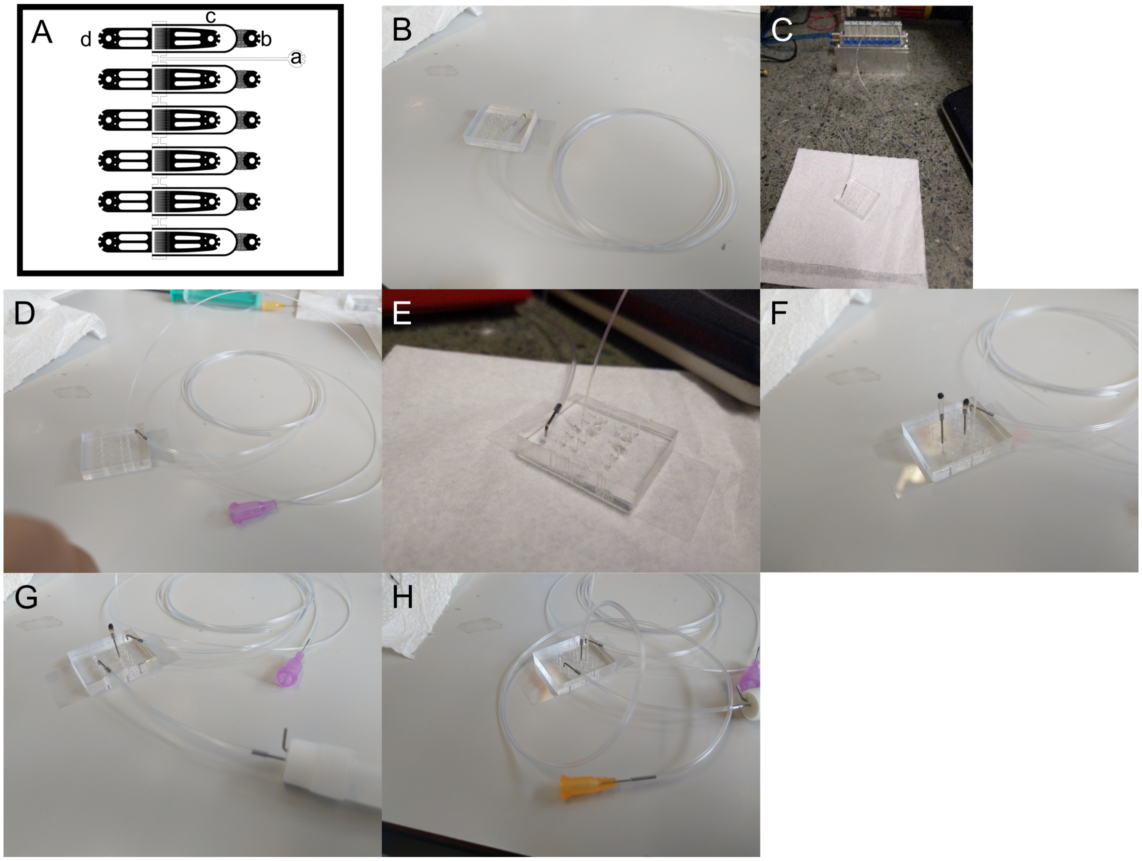

Fig. S2. Device Preparation. (**A)** Schematic device layout. (a) Valve inlet. (b) Food inlet. (c) Worm inlet. (d) Common outlet. (**B**) Connect 1/16” tubing filled with water to (a) using a hollow steel pin. (**C**) Fill on-chip hydraulic valve using the off-chip solenoid (background). Pressurize device for a few minutes. (**D**) Connect syringe with bacteria food to (b) using a piece of 1/32” tubing and a 30G blunt needle. (**E**) Fill device with bacteria food until liquid appears on both open connections. (**F**) Plug (c) and (d) using steel pins blocked with a piece of 1/16” tubing and pressurize the device using the food syringe and its pump. (**G**) Remove the plug from (D) and connect the waste container using a short piece of 1/16” tubing and a hollow steel pin. (**H**) Remove the plug from (c) and connect a buffer-filled syringe to the device using a hollow steel pin and 23G blunt needle. Prior to connecting the tubing draw worms into the tubing and connect the tubing to the device without introducing air into the system

Development • Supplementary information

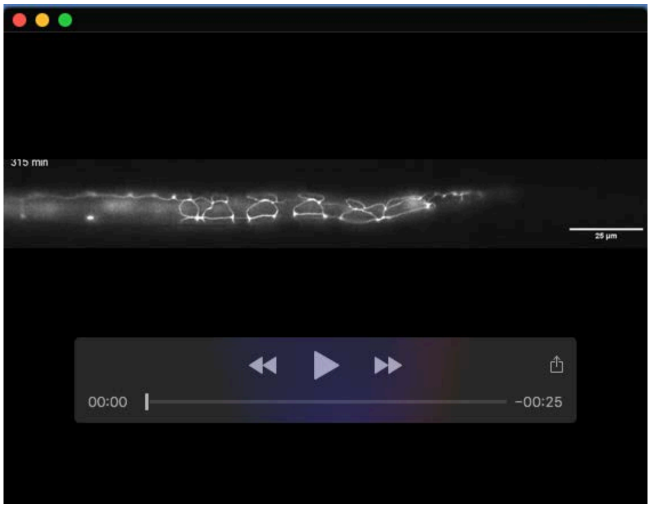

Movie 1. Merged view of two 3D projections of the *C. elegans* vulva during mid L4 stage. Left: epifluorescence images cropped and displayed in unprocessed form. Right: the same stack after deconvolution. Features are outlined using the HMR-1::GFP marker (left and right), and the mCherry::moeABD (right, outlining the AC).

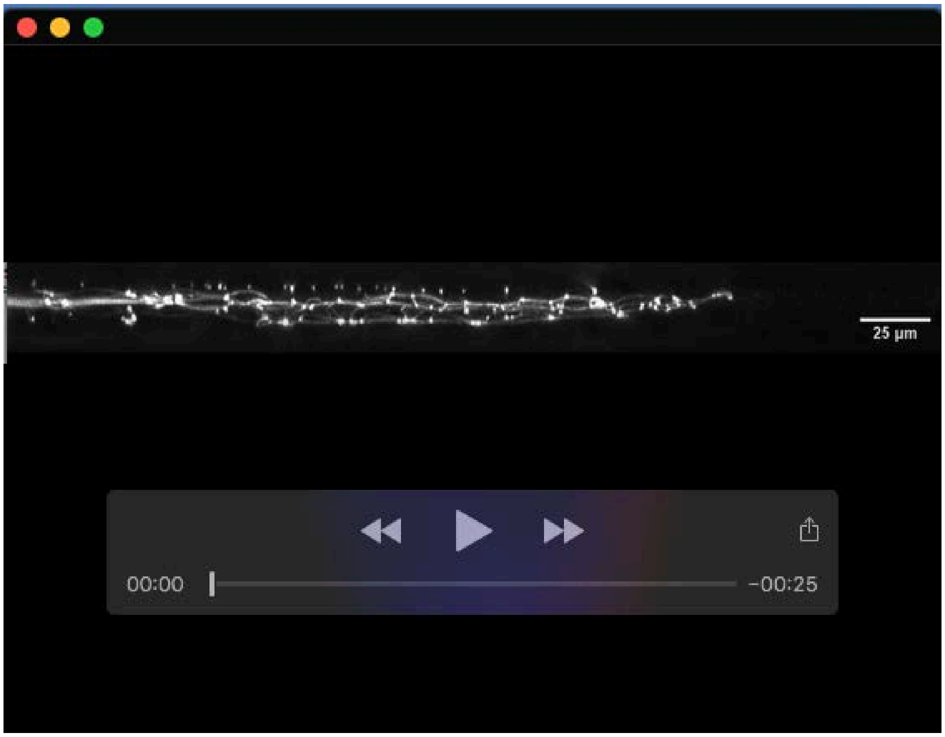

Movie 2. A single *C. elegans* larva developing from overnight starvation up to the early L2 stage. Displayed are maximum intensity projected epifluorescence images (total height of projection is 1.5µm), in otherwise unprocessed format. Visible are both seam cell division and fusion as well as P-cell migration toward the ventral midline and division. Features are outlined using the DLG-1::GFP marker at 15-minute intervals for a total of 25 hours.

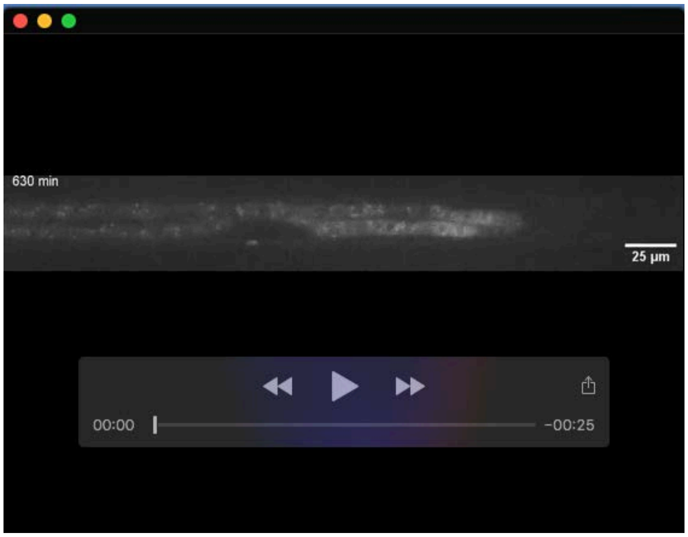

Movie 3. 3D projection of an L1 larvae mid stage. Epifluorescence images deconvolved and brightest points projected along the worm axis. Visible are various seam cells during division and fusion as well the P-cells still attached to the neighboring seam cells, prior to migration. Features are outlined using the DLG-1::GFP marker.

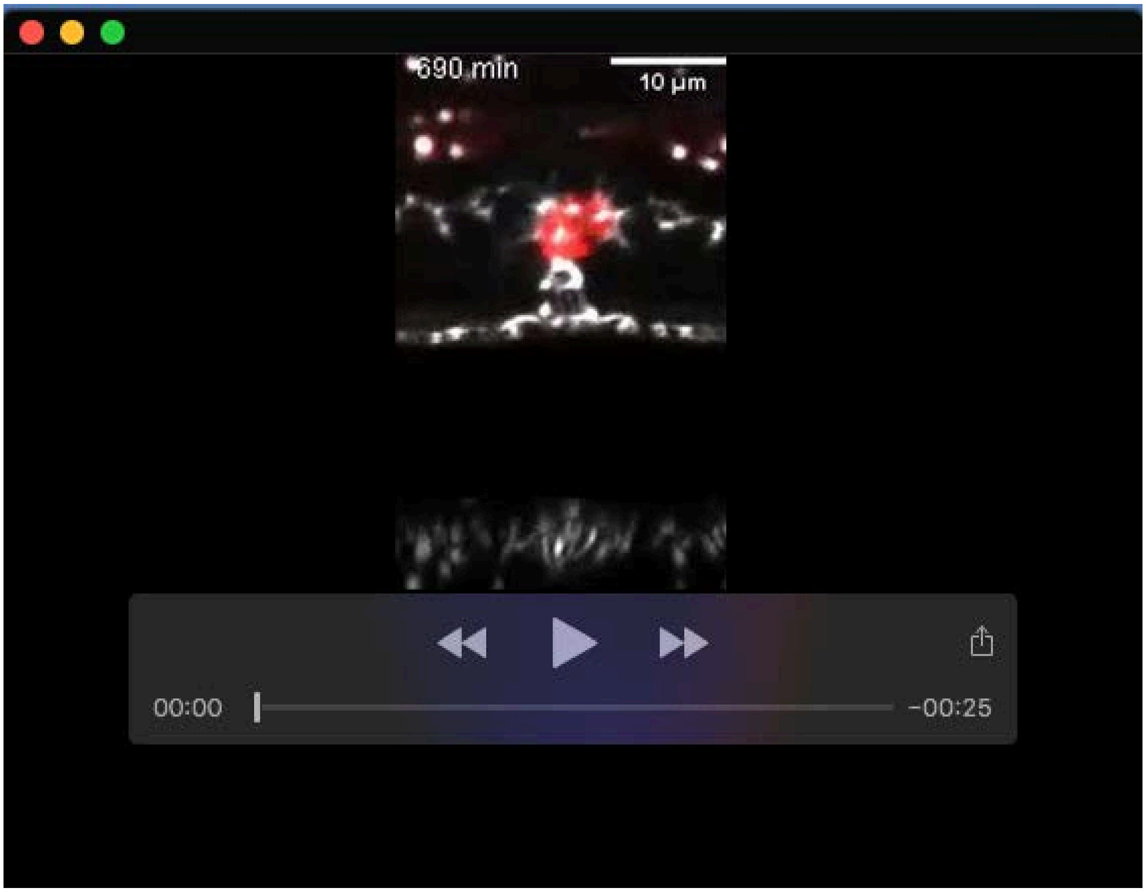

Movie 4. A single larva developing from late L1 stage up to early L4 stage (beginning vulval invagination). Only 1° vulval cells are expressing the EGL-17::CFP marker. In early L2 (approximately 400 minutes), EGL-17::CFP fluorescence manifests in P6.p, continually increasing in intensity. During L3, the 1° fated VPC undergoes two consecutive rounds of division, forming the 1 cell, 2 cell and 4 cell stage of vulva development. All motion visible results from growth as well as slight linear motion still possible on-chip, all of which can easily be removed through image registration. Images are shown at 30-minute intervals for a total of 34 hours.

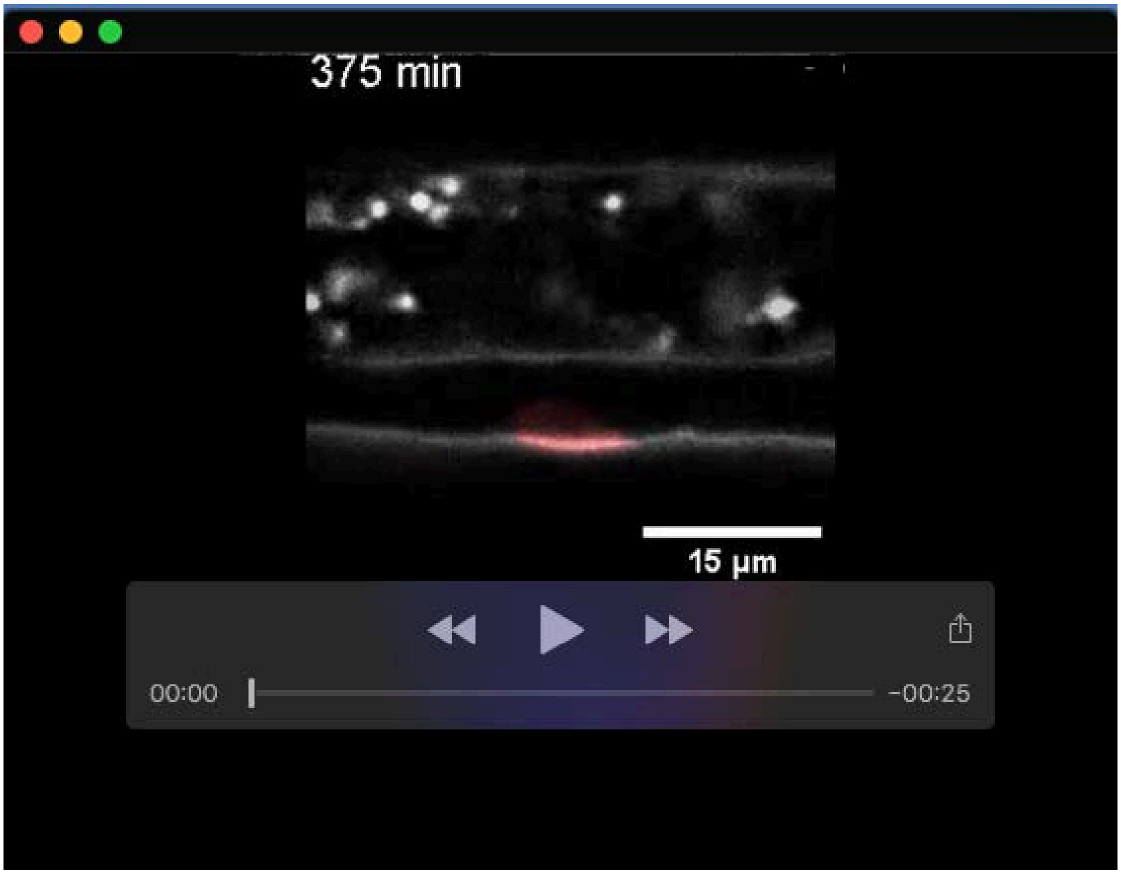

Movie 5. Top view: Z-projection of the developing vulva from the L3 stage up to the L4/adult transition. Bottom view: X-projection of the same vulva over time. Both views show the initial division of the VPCs followed by invagination, formation and enlargement of the toroids followed by collapse during eversion. Epifluorescence images displayed after cropping, deconvolution, registration and projection. Features are outlined through the HMR-1::GFP marker (hypodermis and VPCs) and the mCherry::moeABD marker (outlining the AC).

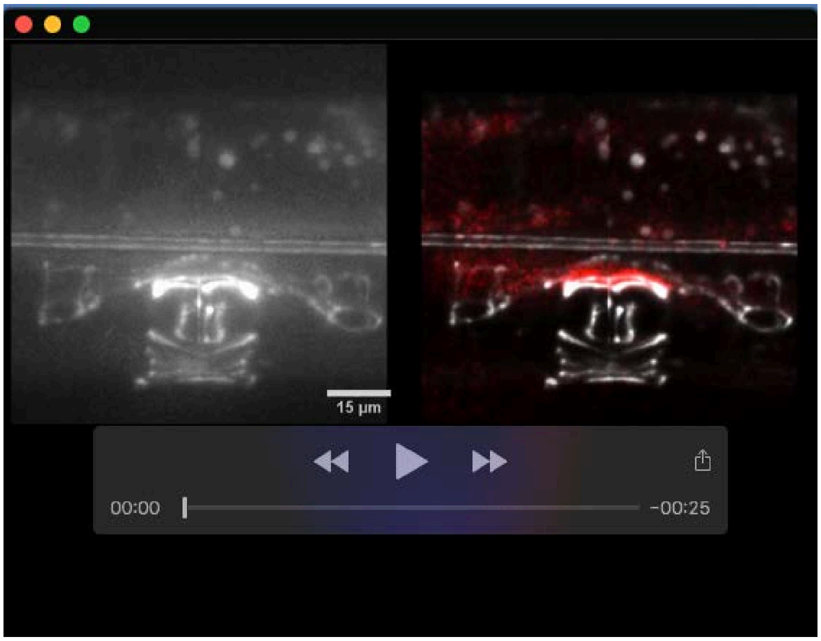

Movie 6. AC invasion in a control animal from L3 to the mid L4 stage. Evident are AC polarization prior to breaching. Features are outlined using the LAM-1::GFP and PIP-2::mCherry marker visualizing the basement membranes and AC respectively. Cropped and deconvolved images shown at 15-minute intervals.

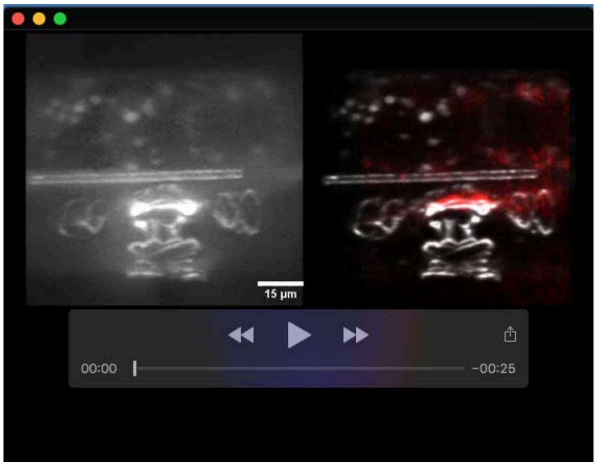

Movie 7. AC invasion in an animal treated with egl-43 RNAi. Evident are multiple ACs, as well as mislocalization of the AC and absence of BM breaching. Features are outlined using the LAM-1::GFP and PIP-2::mCherry marker visualizing the basement membranes and AC respectively. Cropped and deconvolved images shown at 15-minute intervals.

Development • Supplementary information

Supplementary Materials and Methods

Supplementary file 1

CAD file for the L1 device. Four devices with six independent functional units are formed. Note the windows along the midline of the mask, which serve as alignment marker for the following mask. During alignment, the sample is viewed throught the window in this mask and the crosshair formed by the previous mask is placed at the center of the windows formed here.

Click here to Download Supplementary file 1

Supplementary file 2

CAD file for the L1-L4 device. Four devices with six independent functional units are formed. Note the windows along the midline of the mask, which serve as alignment marker for the following mask. During alignment, the sample is viewed throught the window in this mask and the crosshair formed by the previous mask is placed at the center of the windows formed here.

Click here to Download Supplementary file 2

Supplementary file 3

CAD file for the L2-A device. Four devices with six independent functional units are formed. Note the windows along the midline of the mask, which serve as alignment marker for the following mask. During alignment, the sample is viewed throught the window in this mask and the crosshair formed by the previous mask is placed at the center of the windows formed here.

Click here to Download Supplementary file 3

Supplementary file 4

CAD file for the food distribution structure. This structure is the same in all device types and is fabricated first. Once exposed and hardened the structures outlined by this mask are developed and the second layer is spincoated. Note the crosshairs along the midline of the mask, which serve as alignment marker for the following mask. During alignment, make sure there is sufficient overlap between the food channel and the second structure (top and bottom). Overlap on the worm side should not be too large to avoid worms slipping from one channel into the neighboring one, but also not too small to allow sufficient access to food. Aim is an overlap of 20-35µm.

Click here to Download Supplementary file 4

Supplementary file 5

CAD file for the valve layer.

Click here to Download Supplementary file 5

Extended methods with detailed step-by-step protocols

Device Fabrication.

Microfluidic devices for all developmental stages were made from silicon wafers (Si-Wafer 4P0/>1/525±25/SSP/TTV<10, Siegert Wafer, Germany) patterned with SU8 photoresist, following standard protocols protocols (Xia *et al.* 1998). Briefly, in a first step wafers were cleaned with an air plasma (Diener electronic) followed by spin-coating (WS-650-23B, Laurell) the low height, food supply layer, using a low viscosity SU8 (GM1050, Gersteltec). The height of this layer was chosen to be between 3-5µm, high enough for bacteria to pass but low enough for worms to be blocked from entering. This layer was baked and patterned using a first photomask (high-resolution film mask, Microlitho), followed by post-baking and development. In a second step, the higher SU8 layer (8 to 15 µm) was created (GM1050/1060, Gersteltec) baked and exposed through a second photomask. Features created by the first and second mask were aligned using a mask aligner (UV KUB3, Kloe). Following exposure, wafers were post-baked and developed resulting in the final master mold. Prior to use the wafer was baked at 200°C for 10 minutes, resulting in a smooth surface and higher long-term stability of the features. The hard-baked was then treated with chlorotrimethyl silane vapor (Sigma) to passivate the surface.

For device fabrication (Unger *et al.* 2000) the finished master mold was spin-coated with a thin layer of PDMS (spin-coated @750rpm, PDMS Elastosil RT601 A/B, ratio 20:1), while a second batch of PDMS (ratio 5:1) was cast on the master mold with the valve structure (approximately 5mm thick). This master mold was fabricated and treated as described above, however with only a single height layer of SU8. The PDMS on the valve layer was then degassed under vacuum and placed in an oven at 70°C along with the spin-coated device layer. After approximately 15 minutes both PDMS pieces were removed from the oven, the valve layer was carefully removed from the master mold, cut to size and a single access hole punched for each valve structure (G20 puncher, Syneo). The cut PDMS pieces were then aligned to the thin device layer using a stereomicroscope. The two pieces were carefully brought into contact, ensuring proper positioning of the valve on the device and removal of any trapped air. The assembled devices were then placed back into the oven and baked overnight. Following overnight baking, the valve layer and device layer were permanently bonded to one another. At this point all devices are carefully peeled off the master

Development • Supplementary information

mold. All access holes were punched (G20 puncher, Syneo) and devices bonded to cover glass (#41014551, Glaswarenfabrik Karl Hecht) using an air plasma (Zepto, Diener electronic).

Step-by-step protocol

1. Clean wafers using air plasma.
2. Spincoat first SU8 layer, followed by soft-bake at 95°C.
3. Expose first layer, followed by post-bake at 95°C and development.
4. Repeat plasma cleaning after wafer is developed and dry.
5. Spincoat second SU8 layer followed by soft bake at 95°C (if necessary first bake at 65°C).
6. Expose second layer after carefully aligning the second mask to the existing features using alignment markers on either side of the wafer.
7. Post-bake and develop the wafer, followed by hard-bake at 200°C.
8. Treat wafer with chlorotrimethyl silane for at least 2 hours (NOTE: The silanes is toxic and releases corrosive vapors. This step therefore needs to be performed in a fume hood).
9. Prepare a batch of 20g PDMS pre-polymer (Elastosil RT601 part A) and thoroughly mix it with the 1g of crosslinker (ratio 20:1, Elastosil RT601 part B). Ideally mixing is performed using a planetary centrifugal mixer (FlackTek Speedmixer) or if not available by hand using a plastic spatula or glass rod.
10. Remove 2mL of the mixed PDMS using a syringe (note if mixed by hand the PDMS should be degassed prior to removing the necessary part or PDMS should be prepared in a smaller 1-5g batch ratio 20:1).
11. Add another 3.75g of crosslinker (final ratio approx. 5:1).
12. Using the batch of 20:1 PDMS spin coat the device wafer at 750rpm for 30 seconds.
13. Simultaneously place the valve wafer in an aluminium dish and cast the 5:1 PDMS onto it. Degas the device wafer for about 10 minutes and after degassing break all remaining bubbles using pipette.
14. Bake device and valve layer for 15 minutes at 70°C.

Development • Supplementary information

15. Check if device and valve layer are cured sufficiently by poking them with a pipette tip or tweezers. The valve layer should be solid and the device layer should be slightly tacky.
16. Remove the valve layer and cut it to size using a scalpel or razor blade.
17. Punch the access hole to the valve channel.
18. Place the device wafer on a stereomicroscope equipped with top illumination.
19. Gently place the PDMS piece with the valve layer onto the device wafer.
20. Align valve and device features and remove all trapped air from in between the layers.
21. Place assembled device back in the oven at 70°C overnight.
22. Remove the device from the wafer.
23. Punch all remaining access holes.
24. Bond the PDMS to a cover glass using air plasma and place bonded device in the oven at 70°C for a few hours.

Worm Preparation

Worms were bleached from mixed plates, with embryos left to hatch overnight in buffer (M9 or S-Basal). Once hatched worms were filtered through a 10µm cell strainer (#43-10010-50, pluriSelect) to remove all unhatched eggs, as well as debris from the plate or leftover corpses. The L1 larvae were centrifuged at 1300 rcf (in 15mL Falcone tubes) to remove supernatant and washed with 5 mL of clean buffer. Centrifugation was then repeated, supernatant removed and worms transferred to a plate seeded with OP50 if worms older than L1 stage are needed. L1 stage worms can be loaded onto the device right after preparation. Otherwise, once the worms have reached the desired age, they were washed off the plate using S-Basal and left to sediment. The supernatant was then removed and worms washed three times using fresh buffer. Once washed worms were left to sediment, with as much of the supernatant as possible being removed.

Step-by-step protocol

1. Starting from plates with many gravid animals.
2. Wash animals of plate using M9 or S-Basal buffer.
3. Add 5% NaClO and 5M NaOH to the worm suspension (200uL/100uL for every 1mL of worm suspension).

Development • Supplementary information

4. Gently shake bleaching mix until animals begin to break apart.
5. Centrifuge bleaching mix at 1300 rcf for 1 min.
6. Remove supernatant and add an equal amount of fresh buffer.
7. Again centrifuge at 1300 rcf for 1 min.
8. Remove the supernatant and transfer the pelleted worms to a 15mL tube with 5mL of fresh buffer.
9. Shake overnight at 20°C.
10. Filter the worm suspension through a 10µm cell strainer.
11. Centrifuge worm suspension at 1300 rcf for 1 min.
12. Remove supernatant and resuspend worms in an equal amount of buffer.
13. Centrifuge worm mixture again at 1300 rcf for 1 min.
14. Remove supernatant and transfer worms to NGM plates or if L1 larvae are desired, use them right away.
15. Once worms reach desired age, wash them of the plate using fresh S-Basal.
16. Leave worms to sediment by gravity or pellet by centrifugation at 750 rcf.
17. Remove supernatant and add an equal amount of fresh buffer.
18. Repeat sedimentation of centrifugation step.
19. Remove supernatant and add an equal amount of fresh buffer.
20. Repeat sedimentation of centrifugation step for a final time.
21. Remove most of the supernatant.
22. Leave worms in the tube until the experiment starts.

Bacteria preparation

8x5mL of LBroth was prepared in 15mL Falcone tubes, with each tube being inoculated with 0.1mL of NA22 stock (NA22 rather than OP50, as NA22 can be prepared at higher concentrations without clumping. Bacteria were left to grow overnight at 37°C whilst shaking. After growth, the bacteria were centrifuged at 3000 rcf for 2 minutes. The supernatant was then decanted and bacteria re-suspended in 1mL of clean S-Basal (1mL per tube). All tubes were then combined, the centrifugation repeated and three washes performed. Finally all bacteria were centrifuged, decanting as much of the supernatant as possible and re-suspended in 1mL of S-Basal. The concentrated bacteria suspension was mixed with 0.65mL of Optiprep (density matching to prevent

Development • Supplementary information

bacteria from segregating), 0.332mL of S-Basal+1% Pluronic F127. All components were then vortexed and passed through a 10μm cell strainer to remove bacteria clumps formed during the preparation.

Step-by-step protocol

1. Prepare 40mL of LBroth, either as 8x5mL in Falcone tubes or in larger tubes, sterile Erlenmeyer flasks etc.
2. Inoculate LB with 0.1mL of saturated bacteria culture per every 5mL of culture.
3. Grow bacteria while shaking overnight at 37°C.
4. Centrifuge bacteria at 3000 rcf for 2 min.
5. Remove supernatant and add 1mL of fresh buffer to each tube. Combining all tubes into one.
6. Mechanically resuspend bacteria using a pipette.
7. Centrifuge bacteria at 3000 rcf for 2 min.
8. Remove supernatant and again add 2mL of fresh buffer.
9. Mechanically resuspend bacteria using a pipette.
10. Centrifuge bacteria at 3000 rcf for 2 min for a final time.
11. Remove as much of the supernatant as possible and add 1mL of fresh buffer before mechanically resuspending all bacteria.
12. Combine 0.65mL Optiprep, 0.332mL S-Basal+Pluronic F127 with 1mL of bacteria suspension.
13. Mix the food preparation using a vortex mixer.
14. Filter the food through a 10μm cell strainer.

Setup Preparation

For the long-term imaging experiments described above, the system needs to be equipped with a pressure supply and regulator to adjust the pressure exerted on trapped animals by the active on-chip valve. This pressure source must be connected to the off-chip solenoid valve using a sufficient length of tubing and a precision pressure regulator (needed for fine-tuning of the system pressure and stability). The solenoid valve is interfaced to the microfluidic device using a thinner 1/16" tubing. Lastly, the system must be able to actively switch the on-chip valve. The valve is switched

Development • Supplementary information

on 10 seconds before image acquisition, remains active throughout image acquisition and is switched off once all images at a specific time point are acquired. Such active control may be achieved using an Arduino microcontroller and a relay or MOSFET, or in more advanced imaging systems may be integrated with a laser launcher or other DAq card.

- 3/2 solenoid valve (#MH1-A-24VDC-C-HC-V-PS, Festo)
- OD. 6 mm polyurethane tubing (enough to span from the pressure supply to the microscope).
- Precision pressure regulator, 1/4" ports, 2-60psi range, (McMaster #6162K13).
- Pressure gauge, 1/4" connection, 0-60psi range (McMaster #4089K81).
- 2x 1/4" push-in fitting to 1/4" male NPT (McMaster #5111K82).
-

Chip preparation

Refer to Figure 1 and S1. The on-chip hydraulic valve is filled. A long piece of 1/16’’ tubing (#0642002, Tygon tubing 1/16’’, Fisher Scientific) is connected to a 1mL plastic syringe filled with DI water using a blunt needle (#300-35-970, Distrelec), and attached to a microbore steel pin bent at the tip (the pin may be removed from 23G blunt needle). The tubing is connected to the hydraulic valve inlet using the steel pin (**Fig. S2B**). Once connected, the syringe and blunt needle are detached from the tubing and the tubing connected to the valve manifold. The device is pressurized for several minutes until the clamp valve is completely filled with water (1bar pressure) (**Fig. S2C**). Once the valve is filled the pressure is turned off. Next, the device is filled with the bacteria suspension. A long piece of 1/32’’ tubing (#0641900, Tygon tubing 1/32’’, Fisher Scientific) is connected to a 1mL syringe filled with the bacteria food suspension using a 30G blunt needle (#300-35-970, Distrelec) (making sure no air remains in the syringe, needle or tubing). Food is delivered to the chip via the food inlet (**Fig. S2D**), and the device filled with food by gently applying pressure on the syringe plunger, until a drop of liquid appears on each open connection (**Fig. S2E**). All open connections are plugged with a blocked steel pin (attach a small piece of 1/16’’ tubing to a steel pin and burn the end to block it) and more pressure applied to the syringe plunger to remove all air bubbles from the device (**Fig. S2F**). To apply pressure, the setscrew on the back of the syringe pump may be used. Once all air is removed, the blocked steel pins can be

carefully removed and the waste container connected. The waste container is fabricated by gluing several steel pins into the lid of a cryovial and connected to the device using a short piece of 1/16'' tubing and a steel pin (bent 90°) (**Fig. S2G**).

Finally, worms are loaded into the device. Another piece of 1/16'' tubing is connected to a 1mL syringe filled with fresh S-Basal using a blunt needle and steel pin (23G, straight, making sure no air remains in syringe, needle or tubing). The tubing is filled with buffer, and worms "sucked" into the tubing using the syringe. The tubing is connected to the remaining open connection (**Fig. S2H**). Here, a drop of liquid (food) is carefully pushed onto the chip inlet and at the tip of the steel pin (worms). The pin is connected to the chip without introducing air into the device.

Step-by-step protocol

1. Start by filling a syringe with DI water.
2. Attach a 23G needle and a long piece of 1/16" tubing terminated with a hollow steel pin (bent 90°).
3. Fill the tubing with DI water from the syringe and attach it to the valve inlet by pushing the steel pin into the punched hole.
4. Remove the syringe and needle and attach the tubing to the off-chip solenoid.
5. Using the imaging software turn on the solenoid and pressurize the device for several minutes to remove all air from the valve. Check by eye if process complete.
6. Switch off the solenoid.
7. Fill the filtered bacteria solution in a 1mL syringe (approx. 0.5mL of food are sufficient).
8. Attach a 30G needle and a long piece of 1/32" tubing to the needle.
9. Press on the plunger to fill the needle and the attached tubing, ensuring no air stays in the syringe or tubing.
10. Push the 1/32" tubing directly into the food inlet of the microfluidic device (use tweezers ensuring not to damage the tubing, recommended are SMD tweezers).
11. Place the syringe on the syringe pump.
12. Press on the syringe plunger using the mechanical setscrew at the back to fill the device with liquid. A drop of liquid should appear on each open connection.

Development • Supplementary information

13. Plug both the worm inlet and outlet with a blocked steel pin (steel pin with a short piece of 1/16” tubing attached and closed by burning).
14. Apply more pressure using the setscrew to remove all air from within the device.
15. Remove the blocked pin at the outlet and attach the waste container (made from a cryo vial or Eppendorf tube with two steel pins stuck through the lid and held in place by glue). The waste container is attached using a short piece of 1/16” tubing connected to the container on one side and to a hollow steel pin on the other side (bent 90°).
16. Push on the syringe to ensure the waste container is well connected, and no connections blocked.
17. Remove the second blocked steel pin.
18. Push on the syringe until a small drop of liquid appears at the worm inlet.
19. Attach a long piece of 1/16” tubing to a 1mL syringe filled with S-Basal buffer, using a 23G needle.
20. Attach a steel pin (straight) to the other end of the tubing.
21. Fill both needle and tubing with buffer from the syringe ensuring no air stays in the syringe or tubing.
22. Put the steel pin end of the tubing into the tube with worms.
23. Push out a small amount of liquid ensuring no air is left in the tubing.
24. Pull worms into the tubing. Note do not pull them into the syringe.
25. Push on the syringe connected to the worms until a small liquid drop appear on the steel pin.
26. Push the steel pin into the worm inlet.

Worm loading

Worms are loaded into the device channels by gently applying pressure to the syringe plunger, filling as many channels as necessary. Statistically, about 50% of channels should have worms oriented with their head facing the food supply channel. This proportion can be further increased by carefully manipulating the worms while entering the channel. Worms naturally tend to swim against the flow that carries them into the device. For worms to be loaded in headfirst orientation the experimenter should gently push and pull on the worm syringe plunger and wait for animals to turn toward the trap channel. Once oriented correctly worms may be pushed into the device. The

Development • Supplementary information

pump supplying the bacteria suspension is alternatingly run at 1 μL/hr for 30 minutes and 100 μL/h for 5 seconds, looping continuously throughout the experiment (programmable syringe pump Aladdin Al1000-220, WPI).

References

Unger, M.A., Chou, H.P., Thorsen, T., Scherer, A. and Quake, S.R. (2000). Monolithic microfabricated valves and pumps by multilayer soft lithography. *Science*. **288**, 113-116.

Xia, Y. and Whitesides, G.M. (1998). Soft lithography. Annual Review of Materials. *Science*. **28**, 153-184.

Development • Supplementary information
