# Supplementary material for: Microfluidic-based imaging of complete Caenorhabditis elegans larval development
Source: Development. 2021 Jul 21;148(18):dev199674. doi: 10.1242/dev.199674 (PMC8327290; doi:10.1242/dev.199674)
Supplement: Reviewer comments [file develop-148-199674_review_history.pdf]

Microfluidic-based imaging of complete C. elegans larval development

Simon Berger, Silvan Spiri, Andrew deMello and Alex Hajnal
DOI: 10.1242/dev.199674

Editor: Swathi Arur

Review timeline
Original submission: 31 March 2021
Editorial decision: 29 April 2021
First revision received: 2 June 2021
Accepted: 14 June 2021

Original submission

First decision letter

MS ID#: DEVELOP/2021/199674

MS TITLE: Microfluidic-based imaging of complete C. elegans larval development

AUTHORS: Simon Berger, Silvan Spiri, Andrew deMello, and Alex Hajnal

I have now received all the referees reports on the above manuscript, and have reached a decision. The referees' comments are appended below, or you can access them online: please go to BenchPress and click on the 'Manuscripts with Decisions' queue in the Author Area.

The overall evaluation is positive and we would like to publish a revised manuscript in Development, provided that the referees' comments can be satisfactorily addressed. Please attend to all of the reviewers' comments in your revised manuscript and detail them in your point-by-point response. If you do not agree with any of their criticisms or suggestions explain clearly why this is so.

Reviewer 1

Advance summary and potential significance to field

This is an excellent method development paper by Simon Berger et al, in Alex Hajnal's lab where they develop a set of new microfluidic devices that enable observation of C. elegans nematodes during very long timescales, spanning together all of postembryonic development. The quantification of the variability of developmental timing would be very hard to do without a device such as the one presented.

Comments for the author

This is an excellent method development paper by Simon Berger et al, in Alex Hajnal's lab where they develop a set of new microfluidic devices that enable observation of C. elegans nematodes during very long timescales, spanning together all of postembryonic development. The devices enable individual worms to be imaged in parallel, under different magnifications. The demonstrate the utility of the device by imaging a number of cell types throughout the relevant developmental stages: seam cells, P cells, and vulval precursor cells. The quantification of the variability of

© 2021. Published by The Company of Biologists under the terms of the Creative Commons Attribution License (https://creativecommons.org/licenses/by/4.0/).

developmental timing would be very hard to do without a device such as the one presented. They show that the approach is compatible with fluorescence microscopy and produces images of high quality. They also show that they can perform feeding RNAi experiments within the device. They also provide detailed instructions on how to build the devices. I recommend publication.

Minor comments:
- It would be nice if the discussion included consideration on how to adapt these devices for other nematodes, what are the key variables?
- It would be nice to have a discussion, and perhaps a demonstration, of how slow can the processed being imaged need to be. The authors are imaging only a few times per hour. Can the divide be used for faster imaging, for example feeding?
- I could not find the cad files with the device mask. It would be nice if they can provide them.

Reviewer 2

Advance summary and potential significance to field

This manuscript describes a family of microfluidic devices that enable live imaging of C. elegans larval development. These designs isolate multiple worms in a parallel array to continually image the same individuals, together with features for immobilization, feeding to maintain near normal growth and development, and stabilizing their rotational orientation. This system will be useful to C. elegans biologists investigating larval development, and may be a starting point for further designs that allow similar analyses of other worm-like animals with similar dimensions.

Comments for the author

The paper is well written, and the results are clear and detailed. There are no major issues to bring up.
However, there are three medium-level issues that should be addressed or clarified.

1. Details of immobilization.
During immobilization, what is the typical magnitude of movement and duration between movements? Even estimates of this information would be useful, because they may provide approximate limits on how long image acquisition can occur with minimal movement artifacts. The authors benefit from a very nice imaging system with relatively bright markers as an excellent proof of principle.

Knowing these time estimates will help other labs understand if there are any limitations when using a slower imaging system (e.g., with piezo objectives or stages, older CCD), requiring longer exposures (e.g., dim reporters), or imaging smaller objects at higher magnification (e.g., synapses). Are the tails (which are tapered and smaller than the rest of the body) sufficiently immobilized to image? If it is, this would be useful for those studying T cell or male tail development. If not, it would also be good to know of the limitations.

2. On-chip development compared to normal development.
The best justification for the utility of their chip was only left to the end of “Development On-Chip” where the clearest and cleanest comparisons were made on the duration of each larval stage directly compared to on-chip development. This is suitably convincing and builds confidence in the use of this microfluidic chip.

In contrast, during the earlier parts of this section, the authors describe several developmental milestones including L1/L2 moult, P-cell migration and division, seam cell division for animals, and vulval development on-chip, with the timings and the standard deviation of these timings. However without knowing the corresponding values for animals not inside the chip, it is hard to understand the claims made in the paper about normal development. For example, the authors indicate that L2 seam cell divisions occur reproducibly by implying that variability between animals is low (on page 7). This argument can only be supported if variability between animals grown outside the chip is not lower.

© 2021. Published by The Company of Biologists under the terms of the Creative Commons Attribution License (https://creativecommons.org/licenses/by/4.0/).

If the authors want to add the other measurements to support this argument, they should provide numbers and timing for animals are not on the chip for each these events (L1/L2 moult P-cell migration and division seam cell division for animals, and vulval development sub-stages), either from the literature or their own studies with same temperature, so that readers can make the comparison.

If such direct comparisons cannot be made, the authors can still report the values they obtained, and compare them against ranges or estimates of the values for animals off-chip while qualifying their statements.

Temperature is a major factor in developmental rate. The temperatures used during the imaging experiments were not reported, which makes it hard to compare against the literature. If there was no temperature control in the experiments, and worms were just left at an undefined room temperature were the on-plate controls done at approximately the same time so that they were subject to roughly the same temperature and temperature fluctuations?

It is possible that some of the delays are due to slower or variable exit from L1 arrest under starvation, rather than the use of the microfluidic chip and its dimensions.

3. Will the designs for the masks be distributed with the paper for other labs to fabricate chips?

Minor issues:
1. In the introduction, other immobilization methods described in section 2.2 of San-Miguel and Lu Wormbook 2013 should be cited.

2. Based on the images in Figure 4a, 4c, and 4e, the fluorescence values for EGL-17::CFP appear saturated around 120μm gonad length and is therefore no longer indicative of EGL-17 expression levels.

3. It is not clear if the variability in EGL-17 expression is due to the multicopy reporter or actual gene expression noise. I recommend removing the statement about the variability as it is tangential to demonstrating the benefits of using this microfluidic chip.

4. For Figure 3, please indicate the dorsal and ventral sides, the as well as the legend for the arrowhead and asterisks in the insets in 3b-c; these are mentioned in 3d which does not have such labels.

5. There’s a typo on page 7: “Soulston” should be Sulston.

6. In the Supplementary Information, page 7: [REF] should be presumably be substituted with a reference.

<u>Reviewer 3</u>

Advance summary and potential significance to field

This manuscript presents an optimized microfluidic device for imaging C. elegans and specifically illustrates post-embryonic development of hypodermis, vulva morphogenesis, AC invasion and RNAi perturbation. This work shows good improvements on the immobilization, maintenance and long-term imaging of C. elegans. The introduction describing in detail the development of microfluidic devices on the imaging of C. elegans is well-written.

Comments for the author

Minor comments:
In page 5, the length/width of channels for three devices are different especially the length. In the diagram of Fig. 1c, they seem to be of the same size.

© 2021. Published by The Company of Biologists under the terms of the Creative Commons Attribution License (https://creativecommons.org/licenses/by/4.0/).

In page 6, the author mentioned the orientation of loading worms into channels and its statistics (85% correct with head facing the food source and 15% incorrect). Is this related to the stages of worms and devices chosen?

For all of the tests of different developmental events on worm in devices, is it possible to show the development of worms on NGM plates maintained under the same conditions and stages as controls? In page 7, the authors mentioned that the first seam cell division in L2 was typically observed after 21.16±3.5 hours (n = 20) and completion of all L2 divisions after another 12.9±4.0 hours (n = 12). Here the standard error is large. This can also be done in the L1-L4 device which may help fix the data obtained in the L1 device.

In page 7, the authors mentioned that developmental timing of seam cell divisions and fusion to the hypodermis occurred at timing consistent with literature values (Sulston et al. 1983). Is it possible to reproduce the published time as a control in your hands?

In the legend of fig.2, (a'-c') Magnified view of selected time points for images shown in (a-c). Is it possible to label the selections clearly because of too many frames in a-c? Choosing another kind of arrowhead instead of an equilateral triangle may be better.

In Fig. 4a, mark the larval stages in the image. In page 10, the authors mentioned that EGL-17::CFP can also be observed in P5.p and P7.p (Fig. 4a-b).

The images in fig. 4b are not clear. P5.p and P7.p are not shown simultaneously.

It is not clear in Fig. 4d,e why to use time only and not add larval stages gonad length, intensity of marker together. Why do you plot normalized fluorescence over length?

In Fig.5c, what did the linear developmental progression mean?

In the legend of fig.6, the Pcdh-3>mCherry::moeABD reporter mcherry outlines AC.

But in the image, it is PIP-2::mCherry. The contrast of a' and b' can be improved.

The numerical aperture for some lenses is not mentioned (CFI Plan Apo VC 60XC ,
CFI Plan Apo Lambda 60X Oil)

There are also some typos that should be fixed. For example:
In page 3"are worms are"
In page 7 "Soulston"
In page 8 "Sulson"
Some additional general references should be added for example:
Page 9 on seam cells divisions and migrations (Fig. 2):
Austin J & Kenyon C (1994) Cell contact regulates neuroblast formation in the Caenorhabditis elegans lateral epidermis. Development 120:313-324.

Page 9 line 15; Figs. 2-3. The rotation of P cells and seam cell fusions were described in:
Podbilewicz B & White JG (1994) Cell fusions in the developing epithelia of C. elegans. Dev Biol 161(2):408-424.

For timing of Pn.p fusions (Figs 3-4):
Wang BB, et al. (1993) A homeotic gene cluster patterns the anteroposterior body axis of C. elegans. Cell 74:29-42.

Page 11 (Fig. 5). Vulval morphogenesis and connection to uterus:
Newman AP, White JG, & Sternberg PW (1996) Morphogenesis of the C. elegans hermaphrodite uterus. Development 122:3617-3626.

Sharma-Kishore R, White JG, Southgate E, & Podbilewicz B (1999) Formation of the vulva in C. elegans: a paradigm for organogenesis. Development 126:691-699.

© 2021. Published by The Company of Biologists under the terms of the Creative Commons Attribution License (https://creativecommons.org/licenses/by/4.0/).

First revision

Author response to reviewers' comments

Reviewer 1 Comments for the author

It would be nice if the discussion included consideration on how to adapt these devices for other nematodes, what are the key variables?

This aspect was addressed to some extent in the Device Design and Function section. That said, we have now highlighted the role of channel width and height (aiming for a ratio of 0.5-0.6 H/W) in preventing rotation, and defined rules of thumb for choosing the initial channel width (worms should fill 2/3rds of the width at the start of the experiment) and length (which should be as long as the worms are at the end of the experiment) (see "Device Design and Function" line 93-108). Other nematodes are not specifically mentioned, but the same rules will apply.

It would be nice to have a discussion, and perhaps a demonstration, of how slow can the processed being imaged need to be. The authors are imaging only a few times per hour. Can the divide be used for faster imaging, for example feeding?

Faster processes can certainly be imaged. However, a number of factors must be taken into consideration; many of which are not unique to microfluidic environments. Imaging frequency is to some extent limited by phototoxicity and photobleaching, where it becomes a tradeoff between the length of the entire experiment, the desired Z-resolution as well as interval between timepoints. We typically image at 15-minute intervals, since this is a frequent enough for the process we want to study, but not too frequent to cause issues with phototoxicity. For processes, which occur over shorter time periods shorter acquisition intervals are of course possible. Likewise, if very bright markers are used, more frequent imaging becomes simpler. Some of these considerations are discussed in the manuscript, and now emphasized in the revised version; especially limitations that may arise from phototoxicity (see "Development On-Chip" line 207-210 and "Image Acquisition" line 473-485).

I could not find the cad files with the device mask. It would be nice if they can provide them.

Masks designs will now be made available as supplementary information.

Reviewer 2 Comments for the author

Details of immobilization: During immobilization, what is the typical magnitude of movement and duration between movements? Even estimates of this information would be useful, because they may provide approximate limits on how long image acquisition can occur with minimal movement artifacts. The authors benefit from a very nice imaging system with relatively bright markers as an excellent proof of principle. Knowing these time estimates will help other labs understand if there are any limitations when using a slower imaging system (e.g., with piezo objectives or stages, older CCD), requiring longer exposures (e.g., dim reporters), or imaging smaller objects at higher magnification (e.g., synapses).

Using our optical system and markers, we typically observe no motion, and only very rarely motion in the range of 25-50 µm (the most we have observed in vulva morphogenesis, and now added to main text, see "Device Design and Function" line 138-151). This almost certainly due to the high acquisition speed associated with the optical measurement. However, it is interesting to note that we use these devices (via collaboration) on "slower" microscopes, such as spinning disk systems, with excellent results. Some of these results will published in the short-term, further supporting the usefulness of the method presented.

© 2021. Published by The Company of Biologists under the terms of the Creative Commons Attribution License (https://creativecommons.org/licenses/by/4.0/).

Beyond absolute motion during acquisition, other parameters must be considered. If the aim is to create 3D reconstructions of developing features, efficient immobilization becomes crucial in minimizing post processing (see "Device Design and Function" line 138-151). If on the other hand only a single (or few) frame(s) in a stack are relevant (for example in VPC induction or AC invasion) immobilization becomes less critical. Indeed, some of the experiments shown in the manuscript could have been completed without using the on-chip valve at all.

In addition to acquisition speed, a key factor when performing long-term imaging experiments, irrespective of the use of a microfluidic device, is phototoxicity, which puts a natural limit on exposure times, excitation power and imaging intervals relative to the length of a planned experiment. This is aspect is now highlighted in the main text (see "Development On-Chip" line 207-210 and "Image Acquisition" line 473-485) so that readers are aware of all factors that need to be considered.

Are the tails (which are tapered and smaller than the rest of the body) sufficiently immobilized to image? If it is, this would be useful for those studying T cell or male tail development. If not, it would also be good to know of the limitations.

Indeed, the tail and head are less well immobilized on-chip, due to their tapered shape as well as pharyngeal pumping (in case of the head). This is now clearly stated in the manuscript (see "Device Design and Function", line 133-134). Whether or not the device may be used to image these body regions depends again on the imaging requirements. 3D reconstruction of the tail during development will be difficult. However, if only a single image or few slices need to be in focus and aligned, use of the device is perfectly feasible.

On-chip development compared to normal development: The best justification for the utility of their chip was only left to the end of "Development On-Chip" where the clearest and cleanest comparisons were made on the duration of each larval stage directly compared to on-chip development. This is suitably convincing and builds confidence in the use of this microfluidic chip. In contrast, during the earlier parts of this section, the authors describe several developmental milestones including L1/L2 moult, P-cell migration and division, seam cell division for animals, and vulval development on-chip, with the timings and the standard deviation of these timings. However, without knowing the corresponding values for animals not inside the chip, it is hard to understand the claims made in the paper about normal development. For example, the authors indicate that L2 seam cell divisions occur reproducibly by implying that variability between animals is low (on page 7). This argument can only be supported if variability between animals grown outside the chip is not lower. If the authors want to add the other measurements to support this argument, they should provide numbers and timing for animals are not on the chip for each these events (L1/L2 moult, P-cell migration and division, seam cell division for animals, and vulval development sub-stages), either from the literature or their own studies with same temperature, so that readers can make the comparison. If such direct comparisons cannot be made, the authors can still report the values they obtained, and compare them against ranges or estimates of the values for animals off-chip while qualifying their statements.

We have added our own on-plate measurements for seam cell fusion and P-cell migration (post-embryonic development of the hypodermis), with generally good agreement with the results found on chip (see "Post-embryonic development of the hypodermis" line 246-252). Furthermore, we have now included a clear comparison of our on- and off-chip results for this process to the results reported by Podbilewicz et al., with again generally good agreement, except for a slight developmental delay toward the end of the L1 stage (see "Post-embryonic development of the hypodermis" line 246-252). Similarly, we now include a clear comparison of the developmental time found for vulva development (specifically the transition between distinct substages of vulva formation) with the results presented by Mok et al. (see "Live-imaging vulval morphogenesis" line 323-326). Likewise, for VPC induction and AC invasion, literature comparison was included, looking at the specific time by which the first and second P.6p division occurs ("Observing VPC induction" line 280-285) and the time over which AC invasion occurs ("On-Chip RNAi Perturbation of AC Invasion" line 351-355). In either case, we found good agreement of the observed timing on-chip and on NGM plates.

© 2021. Published by The Company of Biologists under the terms of the Creative Commons Attribution License (https://creativecommons.org/licenses/by/4.0/).

Unfortunately, providing comparable results from worms grown off-chip, especially with appropriate resolution and at the single individual level, is not feasible in our hands. We therefore hope that the clear comparison to general developmental timing in the literature will suffice and allow readers to judge the presented method.

Temperature is a major factor in developmental rate. The temperatures used during the imaging experiments were not reported, which makes it hard to compare against the literature. If there was no temperature control in the experiments, and worms were just left at an undefined room temperature, were the on-plate controls done at approximately the same time so that they were subject to roughly the same temperature and temperature fluctuations?

Temperature for all experiments (on- an off-chip) was maintained at 20±0.5°C. This was accomplished either using ambient temperature control or a microscope incubator. In the case of plate experiments, NGM plates were incubated at 20°C and worms only removed from the incubator when preparing agar pads. Relevant passages on experiment temperature have been added to the Image Acquisiton section of Materials and Methods (see "Image Acquisiton" line 493-495).

It is possible that some of the delays are due to slower or variable exit from L1 arrest under starvation, rather than the use of the microfluidic chip and its dimensions.

L1 arrest certainly contributes to some of the observed variation. We generally time L1 starvation very precisely, such that worms for different experiments are grown in a highly consistent manner. This limits any such influences. However, as delays were typically observed more strongly on-chip, a contribution of the chip to such delays seems likely. In addition to the delay arising from the device, phototoxicity may be an issue. Unlike off-chip worms, on-chip worms are frequently imaged, which may cause additional stress and delay, even though illumination is kept to a minimum. We have now expanded this discussion in the main text (see "Development On-Chip" line 207-210).

3. Will the designs for the masks be distributed with the paper for other labs to fabricate chips?

Masks designs will now be made available as supplementary information.

In the introduction, other immobilization methods described in section 2.2 of San-Miguel and Lu, Wormbook 2013 should be cited.

A selection of citations has been added to the Introduction section.

Based on the images in Figure 4a, 4c, and 4e, the fluorescence values for EGL-17::CFP appear saturated around 120µm gonad length and is therefore no longer indicative of EGL-17 expression levels.

Indeed, at approximately 120µm the fluorescence signal seems to have plateaued. This however is not saturation of the raw signal. In the raw images for the 4 cell stages of vulval development, the cells show an average maximum intensity of ~1000 counts; far from the maximum intensity that our cameras can register. The cells in the referenced images appear saturated solely due to the chosen contrast. Contrast values were chosen such that the primary VPC would be visible throughout all timeframes of the image (4a). As cells are dim early on, they appear bright later in the experiment.

It is not clear if the variability in EGL-17 expression is due to the multicopy reporter or actual gene expression noise. I recommend removing the statement about the variability as it is tangential to demonstrating the benefits of using this microfluidic chip.

We opted to leave the argument on variability of EGL-17 expression in the manuscript. EGL-17 expression has been shown to vary early on during development, occurring not only in P6.p. Being able to quantify such variation, as well as the large animal to animal variation observed throughout the experiment in vivo certainly underlines the methods utility in studying comparable dynamic systems or population heterogeneity.

© 2021. Published by The Company of Biologists under the terms of the Creative Commons Attribution License (https://creativecommons.org/licenses/by/4.0/).

For Figure 3, please indicate the dorsal and ventral sides, the as well as the legend for the arrowhead and asterisks in the insets in 3b-c; these are mentioned in 3d which does not have such labels.

A label for the asterisks and an arrowhead have been added for 3b-c and removed for 3d. All images now face their ventral side down, which is indicated in the figure legend (see Fig.3 legend, line 657).

There's a typo on page 7: "Soulston" should be Sulston.

This typo has been corrected.

In the Supplementary Information, page 7: [REF] should be presumably be substituted with a reference.

The [REF] has been removed and replaced by the corresponding references (i.e. Xia et al. and Unger et al.).

Reviewer 3 Comments for the author

In page 5, the length/width of channels for three devices are different, especially the length. In the diagram of Fig. 1c, they seem to be of the same size.

The length and width of the channel refer to the section from the tip to narrowing (the effective trap region), not the entire length of the channel. In Fig.1c we originally showed the imaging region as part of the full channel length. This appears to have caused some confusion. Accordingly, we have revised the figure, cutting the channels in 1c at the narrowing, and removing the upstream channel section.

In page 6, the author mentioned the orientation of loading worms into channels and its statistics (85% correct with head facing the food source and 15% incorrect). Is this related to the stages of worms and devices chosen?

No, this is irrespective of stage. Head/tail orientation is entirely dependent on the worms and their particular behavior. Worms have a natural tendency to swim against the flow that carries them into the device and therefore slightly favor being loaded in a "tail first" orientation. However, through manual manipulation, worms can be oriented headfirst and then pushed into the channel. Manipulation primarily consists of gentle pushing and pulling on the worm syringe plunger and waiting for a specific worm to turn its head toward the channel before pushing it in. This works well for most animals but some animals will still slip in tail first. A brief explanation on the manual manipulation has been added to the Supplementary Information in the Worm Loading section (see "Worm Loading" p.15, SI).

For all of the tests of different developmental events on worm in devices, is it possible to show the development of worms on NGM plates maintained under the same conditions and stages as controls?

Unfortunately, showing the same processes in the same detail will be almost impossible off-chip as specific animals cannot be tracked. Instead, we have added clear comparison to literature on developmental timing wherever possible (see response to Reviewer 2).

In page 7, the authors mentioned that the first seam cell division in L2 was typically observed after 21.16±3.5 hours (n = 20) and completion of all L2 divisions after another 12.9±4.0 hours (n = 12). Here the standard error is large. This can also be done in the L1-L4 device which may help fix the data obtained in the L1 device.

Indeed, far better results in L2 development can be obtained with the L1-L4 device rather than the L1 device. The data referred to were included to illustrate the developmental delays

© 2021. Published by The Company of Biologists under the terms of the Creative Commons Attribution License (https://creativecommons.org/licenses/by/4.0/).

mentioned in the text. Illustrating that studying development from early L1 to L2 stage in this device is possible, although not recommended.

In page 7, the authors mentioned that developmental timing of seam cell divisions and fusion to the hypodermis occurred at timing consistent with literature values (Sulston et al. 1983). Is it possible to reproduce the published time as a control in your hands?

Here, we have added new results from on plate experiments, assessing the timing of seam cell division (the first and last) as well as fusion and P-cell migration/division. These results compare well with on-chip data. We have also added a clear comparison between literature values and our experimental results on- and off-chip. (see "Post-embryonic development of the hypodermis" line 243-252)

In the legend of fig.2, (a'-c') Magnified view of selected time points for images shown in (a-c). Is it possible to label the selections clearly because of too many frames in a-c? Choosing another kind of arrowhead instead of an equilateral triangle may be better.

Time points from which images in a'-c' were taken have now been marked in a-c by an asterisk at the upper right corner of each worm image. Arrowheads in a'-c' (as well as in all other figures) have been reworked.

In Fig. 4a, mark the larval stages in the image.

Markers for larval stage have been added.

In page 10, the authors mentioned that EGL-17::CFP can also be observed in P5.p and P7.p (Fig. 4a-b). The images in fig. 4b are not clear. P5.p and P7.p are not shown simultaneously.

Typically expression of EGL-17::CFP is observed in P6.p and sometimes in either P5.p or P7.p, never in both. This statement has therefore been modified in the main text to "may also be observed in the neighboring VPCs" as we only show P5.p (see "Observing VPC induction" line 275-276 and Fig.4 legend, line 664-666). Indeed, fluorescence in the VPCs at the stage shown in 4b is low compared to background. Nonetheless, faint fluorescence in P6.p and P5.p can be observed, with the fluorescence in P5.p disappearing over time. Better images could not be acquired, as that would result in high phototoxicity and therefore developmental arrest.

It is not clear in Fig. 4d,e why to use time only and not add larval stages, gonad length, intensity of marker together. Why do you plot normalized fluorescence over length?

We used gonad length as a measure of reproducible development in 4d. Time in 4d is experimental time, with time 0 being the start of the experiment and not developmental worm age. When time refers to experimental time is clarified in the figure text (see Fig.4 legend, line 668-674). In 4e we use gonad length instead of experiment time to get a common starting point for all imaged animals, eliminating possible differences resulting from different developmental age.

In Fig.5c, what did the linear developmental progression mean?

We simply took the linear increase in feature size as an indicator of continued development, assuming that developmental delays, large variation between animals and most certainly developmental arrest would result in a deviation from such a linear progression. This conclusion has been added to the relevant figure caption. (see Fig.5 legend, line 692-693)

In the legend of fig.6, the Pcdh-3>mCherry::moeABD reporter mcherry outlines AC. But in the image, it is PIP-2::mCherry.

This was an error and has now been corrected. The used marker was mCherry::moeABD.

The contrast of a' and b' can be improved.

© 2021. Published by The Company of Biologists under the terms of the Creative Commons Attribution License (https://creativecommons.org/licenses/by/4.0/).

Here, achieving better contrast is difficult due to the low signal-to-noise ratio of the LAM-1::GFP marker, in conjunction with the bright gut granules in the background. Images could be exchanged for deconvolved ones, which indeed offer better signal to noise ratio. However, we feel the image quality is sufficient to make the point that we want to make, and generally think the readers can more objectively judge the capabilities of the devices if we show the achievable raw image quality without post-processing.

The numerical aperture for some lenses is not mentioned (CFI Plan Apo VC 60XC , CFI Plan Apo Lambda 60X Oil).

Numerical aperture has been added for the two lenses in question.

There are also some typos that should be fixed. For example: In page 3“are worms are”, In page 7 “Soulston”, In page 8 “Sulson”.
These typos have been corrected.

Some additional general references should be added.

The suggested references were added to the relevant parts of the text.

Page 9 on seam cells divisions and migrations (Fig. 2):
Austin J & Kenyon C (1994) Cell contact regulates neuroblast formation in the Caenorhabditis elegans lateral epidermis. Development 120:313-324.
Page 9 line 15; Figs. 2-3. The rotation of P cells and seam cell fusions were described in:
Podbilewicz B & White JG (1994) Cell fusions in the developing epithelia of C. elegans. Dev Biol 161(2):408-424.
For timing of Pn.p fusions (Figs 3-4):
Wang BB, et al. (1993) A homeotic gene cluster patterns the anteroposterior body axis of C. elegans. Cell 74:29-42.
Page 11 (Fig. 5). Vulval morphogenesis and connection to uterus:
Newman AP, White JG, & Sternberg PW (1996) Morphogenesis of the C. elegans hermaphrodite uterus. Development 122:3617-3626.
Sharma-Kishore R, White JG, Southgate E, & Podbilewicz B (1999) Formation of the vulva in C. elegans: a paradigm for organogenesis. Development 126:691-699.

Second decision letter

MS ID#: DEVELOP/2021/199674

MS TITLE: Microfluidic-based imaging of complete C. elegans larval development

AUTHORS: Simon Berger, Silvan Spiri, Andrew deMello, and Alex Hajnal
ARTICLE TYPE: Techniques and Resources Article

I am happy to tell you that your manuscript has been accepted for publication in Development, pending our standard ethics checks.

Reviewer 2

Advance summary and potential significance to field

The advances in this paper and its significance remains the same as the first round of review.

Comments for the author

© 2021. Published by The Company of Biologists under the terms of the Creative Commons Attribution License (https://creativecommons.org/licenses/by/4.0/).

I thank the authors for addressing all the essential points that I raised during the review. I enjoyed reading this manuscript. In particular I, very much appreciate the efforts made to compare developmental rates against literature values.

Reviewer 3

Advance summary and potential significance to field

The authors have answered all the suggestions from the reviewers.

Comments for the author

The manuscript is suitable for publication.

© 2021. Published by The Company of Biologists under the terms of the Creative Commons Attribution License (https://creativecommons.org/licenses/by/4.0/).
